# Reply to Roepe, “The collective creativity of Drs. Baro, Pooput, and Callaghan”

**DOI:** 10.1128/aac.00153-26

**Published:** 2026-03-16

**Authors:** Iqbal Taliy Junaid, Ashutosh Panda, Arunaditya Deshmukh, Rahila Sardar, Monika Narwal, Prakhar Agrawal, Neha Prakash, Asif Akhtar, Amit Kumar Dey, Suneet Shekhar Singh, Saptarshi Mridha, Jigneshkumar Mochi, Sadaf Parveen, Mohit Kumar, Rashi Nagar, Naseem Gaur, Dinesh Gupta, Asif Mohmmed, Inderjeet Kaur, Krishanpal Karmodiya, Pawan Malhotra

**Affiliations:** 1 Malaria Biology Group, International Centre for Genetic Engineering and Biotechnology (ICGEB) 28845 , New Delhi, India; 2 Translational Bioinformatics Group, International Centre for Genetic Engineering and Biotechnology 28845, New Delhi, India; 3 Parasite Cell Biology Group, International Centre for Genetic Engineering and Biotechnology (ICGEB) 28845 , New Delhi, India; 4 Department of Biology, Indian Institute of Science Education and Research 193158, Pune, Maharashtra, India; 5 Yeast Biofuel Group, International Centre for Genetic Engineering and Biotechnology 28845, New Delhi, India; 6 Department of Biotechnology, Central University of Haryana 242287, Mahendergarh, Haryana, India; The Children's Hospital of Philadelphia, Philadelphia, Pennsylvania, USA

## REPLY

We acknowledge Dr. Paul D. Roepe’s concern regarding citation of earlier foundational studies describing codon-optimized back-translation of *Plasmodium* transporter genes, heterologous expression in *Saccharomyces cerevisiae*, and drug susceptibility evaluation using yeast spotting and growth assays, which were pioneered and refined by his laboratory (1). We fully agree that these contributions represent important methodological advances in transporter functional studies, and we appreciate the opportunity to recognize this prior work explicitly (2–4).

Dr. Roepe also notes that PfAAT1 (a digestive vacuole-localized transporter implicated in chloroquine resistance evolution) was not detected or discussed in our study. We appreciate this observation and acknowledge that proteome-based detection of FV-resident proteins can be influenced by multiple factors, including abundance, extraction efficiency, membrane association strength, and technical limitations of the purification workflow. However, it was not captured in our data set by both the methods.

We understand his concern and believe that these additions will be useful for the next set of studies for understanding the link between other transporters and drug resistance.
